# The associations between peripheral inflammatory and lipid parameters, white matter hyperintensity, and cognitive function in patients with non-disabling ischemic cerebrovascular events

**DOI:** 10.1186/s12883-024-03591-6

**Published:** 2024-03-04

**Authors:** Binghan Li, Zhengsheng Gu, Weisen Wang, Bingying Du, Chenghao Wu, Bin Li, Tianren Wang, Ge Yin, Xin Gao, Jingjing Chen, Xiaoying Bi, Hailing Zhang, Xu Sun

**Affiliations:** Department of Neurology, Shanghai Changhai Hospital, The Second Military Medical University, Shanghai, China

**Keywords:** Non-disabling ischemic cerebrovascular event, White matter hyperintensity, Lipid Parameter, Vascular cognitive impairment, Low density lipoprotein-C, Serum biomarker

## Abstract

**Background:**

The global prevalence of VCI has increased steadily in recent years, but diagnostic biomarkers for VCI in patients with non-disabling ischemic cerebrovascular incidents (NICE) remain indefinite. The primary objective of this research was to investigate the relationship between peripheral serological markers, white matter damage, and cognitive function in individuals with NICE.

**Methods:**

We collected clinical data, demographic information, and medical history from 257 patients with NICE. Using the MoCA upon admission, patients were categorized into either normal cognitive function (NCF) or VCI groups. Furthermore, they were classified as having mild white matter hyperintensity (mWMH) or severe WMH based on Fazekas scores. We then compared the levels of serological markers between the cognitive function groups and the WMH groups.

**Results:**

Among 257 patients with NICE, 165 were male and 92 were female. Lymphocyte count (OR = 0.448, *P* < 0.001) and LDL-C/HDL-C (OR = 0.725, *P* = 0.028) were protective factors for cognitive function in patients with NICE. The sWMH group had a higher age and inflammation markers but a lower MoCA score, and lymphocyte count than the mWMH group. In the mWMH group, lymphocyte count (AUC = 0.765, *P* < 0.001) and LDL-C/HDL-C (AUC = 0.740, *P* < 0.001) had an acceptable diagnostic value for the diagnosis of VCI. In the sWMH group, no significant differences were found in serological markers between the NCF and VCI groups.

**Conclusion:**

Lymphocyte count, LDL-C/HDL-C were independent protective factors for cognitive function in patients with NICE; they can be used as potential biological markers to distinguish VCI in patients with NICE and are applicable to subgroups of patients with mWMH.

## Introduction

Vascular cognitive impairment (VCI) is a group of disorders characterized by cognitive impairment caused by vascular etiologies, accounting for approximately 30 − 40% of all cases of cognitive impairment [1, 2]. It is the second most prevalent form of cognitive impairment, after Alzheimer’s disease (AD) [3]. 

The presence of VCI is intricately linked to cerebrovascular diseases (CVD), as evidenced by various recent studies highlighting the role of blood-brain barrier dysfunction, cerebral ischemia hypoxia, and abnormal lipid metabolism in the central nervous system as consequences of CVD. These factors contribute to persistent inflammatory responses and subsequent white matter injury [4–6]. White matter hyperintensities (WMHs), recent small subcortical infarcts, cerebral microbleeds and perivascular spaces are all unique neuroimaging markers of VCI, in which WMHs are more able to reflect white matter injury [7]. In our prior investigation, we established a positive correlation between the severity of WMHs and the extent of cognitive decline in individuals afflicted with CVD [8].

Several studies have shown that cholesterol levels in the central nervous system are closely related to cognitive function [9, 10]. On one hand, the close association between cholesterol and various aspects of brain function, such as cytotropic support, neuronal activity, and neural information transmission, highlights its significance as a crucial component of neurons [11, 12]. However, on the other hand, cholesterol abnormalities are also implicated in the development of atherosclerosis, chronic neuroinflammatory responses, and endothelial cell dysfunction, thereby serving as a risk factor for CVD. Nevertheless, the precise connection between cholesterol and VCI remains a topic of substantial debate [13, 14]. Recent research has demonstrated that neuroinflammation serves as a mediator in both lipid metabolism and cognitive function. Furthermore, it has been suggested that inflammation and lipid-related markers in peripheral blood may provide some insight into the state of central system inflammation and lipid metabolism [15, 16]. However, the usefulness of these markers in predicting VCI remains a topic of debate. Existing studies primarily focus on elucidating the association between peripheral blood lipid or inflammatory indicators and cognitive function, with limited exploration of the diagnostic value of varying degrees of white matter injury [17, 18]. 

Lymphocytes, neutrophils, LDL-C and HDL-C are frequently utilized as peripheral blood markers of systemic inflammation and systemic lipid levels, with indications suggesting that these markers may effectively hold promise as diagnostic indicators for VCI [19, 20]. Meanwhile, compared with other cerebrospinal fluid markers or special plasma proteins, the above indicators can be obtained more economically and safely in clinic [21]. 

Non-disabling ischemic cerebrovascular events (NICE) are prevalent in the Chinese population and are considered the most common type of CVD [22]. In order to contribute to the identification of potential biomarkers for the diagnosis of VCI, this study aims to examine the associations between inflammatory and lipid markers in peripheral blood, white matter injury, and cognitive function in patients with NICE.

## Materials and methods

### Research participants

We consecutively enrolled and collected clinical data for 270 patients with NICE treated at Changhai Hospital (Shanghai, China) between January 2021 and October 2022. Among them, thirteen patients who had not undergone head MRI were excluded; therefore, 257 patients were included in the final analysis. The inclusion criteria for patients with NICE were as follows: (a) first episode of NICE; (b) age 18–80 years; (c) National Institute of Health Stroke Scale Score (NIHSS) ≤ 3 at admission; (d) acute phase stroke with admission within 1 week of onset; and (e) no or only mild neurological deficits not exerting substantial effects on daily life or functioning.

The following were the exclusion criteria: (a) inability to effectively complete the cognitive assessment (e.g., global aphasia, severe vision impairment, etc.); (b) presence of cognitive impairments other than VCI (c) history of organic mental disorder; (d) severe liver or kidney dysfunction; (f) history of inflammatory or autoimmune diseases; (e) history of malignant tumor; and (g) refusal to participate in the study. All patients underwent clinical evaluation and diagnosis according to the guidelines and underwent standardized treatment according to the specifications.This study was approved by the Ethics Committee of Changhai Hospital (CHEC2022-227), and each participant gave written informed permission.

### Scores of WMH

T1-weighted (T1WI), T2-weighted (T2WI), and T2-weighted fluid-attenuated inversion recovery (T2WI-FLAIR) sequences were used for head MRI on all patients. Based on the location of hyperintensities in the deep white matter and paraventricular regions on T2WI, WMHs were categorized using the Fazekas scale (0, normal; 1, mild; 2, moderate; 3, severe). Mild WMH (mWMH) was described as a Fazekas score of 0 or 1, and severe WMH (sWMH) as a Fazekas score of 2 or 3. Two qualified neurologists independently interpreted the imaging data. Conflicts were settled by a third senior doctor.

### Assessment of neurocognition

The Montreal Cognitive Assessment (MoCA) scale was used to evaluate the cognitive function of the patients. Every assessor received training in standardizing the scale’s scoring. MOCA scores of fewer than 24 were used to determine the VCI group. Numerous studies based on the Chinese population indicate MoCA’s strong specificity and sensitivity when the critical score is 24 [23, 24]. Consequently, individuals were classified as having cognitive impairment if their overall score was less than 24. Every patient enrolled in this study had an acute ischemic stroke, and every patient in the cognitive impairment group met the VCI criteria with a Hachinski Ischemic Scale of more than 7. We employed the following measures, which have been demonstrated to be useful for the diagnosis of VCI, to further evaluate the cognitive domain: working memory capacity was measured with the Digit Span Test (DST), Stroop Color-Word Test (SCWT), Symbol Digit Modalities Test (SDMT), Auditory Verbal Learning Test (AVLT), and Trail Making Test A/B (TMB-A/B) [25]

### Statistical analysis

For the statistical studies, SPSS 26.0 (IBM, U.S.) was utilized. Measurement data with normal distributions (Shapiro-Wilk Test) were expressed as mean ± standard deviation, and comparisons between groups were performed using independent samples t-tests or analyses of variance. Measurement data with a non-normal distribution were expressed as median (lower quartile, upper quartile), and comparisons between groups were performed using the Mann-Whitney U-test. The χ2 test was used to compare groups based on enumeration data that were reported as numbers and percentages.

The relationship between multiple biomarkers and cognitive performance in patients with CVD was examined using a binary logistic regression model. Partial indexes will be screened in a collinearity diagnosis in the regression model. Furthermore, receiver operating characteristic (ROC) curves concerning the diagnostic significance of relevant serological markers for VCI were produced using patients with normal cognitive function (NCF) as the reference group following grouping based on WMH.

## Results

### Grouping

A cohort of 257 individuals diagnosed with NICE, consisting of 165 males and 92 females, were enrolled in the study. Among them, 125 patients belonged to the NCF group while 132 were classified under the VCI group. Furthermore, the sWMH group encompassed 111 patients, whereas the mWMH group included 146 individuals, as determined by their Fazekas scores.

### Comparisons between the mWMH and sWMH groups

Patients in the sWMH group were found to be significantly older compared to those in the mWMH group (*P* < 0.001). Additionally, the sWMH group exhibited lower scores on the MoCA, AVLT, SDMT, SCWT, and DST (in reverse order) in comparison to the mWMH group (all *P* < 0.001). Furthermore, the sWMH group displayed reduced levels of lymphocyte count, serum iron level, and glomerular filtration rate (all *P* < 0.05) when considering serological indicators. Conversely, the sWMH group demonstrated significantly higher neutrophil count, MLR, PLR, SII, SIRI, Lipoprotein(a), and NLR than the mWMH group (all *P* < 0.05). Notably, there were no significant differences observed between the two groups in terms of sex, BMI, history of diabetes, hypertension, smoking, alcohol intake, or other serological markers. (Table 1)

**Table 1:** Comparison of variables between the sWMH and mWMH groups

Characteristics	sWMH (*n* = 111)	mWMH (*n* = 146)	P value	Cohen’s d
Age (years)	67.00(58.00,73.00)	61.00(54.00,68.00)	**< 0.001**	**0.069**
BMI (kg/m^2^)	24.48 ± 3.35	24.38 ± 3.56	0.422	0.103
Gender, male, n (%)	72(64.8%)	93(63.7%)	0.950	
Hypertension, n (%)	83(74.8%)	96(65.8%)	0.155	
Diabetes, n (%)	40(36.0%)	42(28.8%)	0.269	
Smoking, n (%)	45(40.5%)	68(46.6%)	0.354	
Alcoholism, n (%)	33(29.7%)	48(32.9%)	0.738	
Lymphocyte (* 10 ^**9**^ **/L)**	**1.69 ± 0.48**	**1.96 ± 0.0.65**	**< 0.001**	**0.455**
Neutrophil (* 10 ^**9**^ **/L)**	**3.86(3.30,4.31)**	**3.45(2.60,4.51)**	**0.029**	**0.201**
Platelet (* 10^9^/L)	203.0 ± 48.64	210.0 ± 55.89	0.669	0.067
**NLR**	**2.34(1.85,3.07)**	**1.86(1.45,2.46)**	**< 0.001**	**0.349**
**MLR**	**0.27(0.23,0.34)**	**0.24(0.21,0.31)**	**0.003**	**0.350**
**PLR**	**126.31 ± 46.65**	**119.02 ± 44.64**	**0.006**	**0.310**
**SII**	**475.58(359.18,585.93)**	**350.30(291.27,522.41)**	**< 0.001**	**0.285**
**SIRI**	**1.03(0.80,1.39)**	**0.85(0.59,1.28)**	**< 0.001**	**0.319**
WBC (* 10^9^/L)	6.32(5.44,7.24)	6.30(4.91,7.73)	0.430	0.065
**GFR (mL/min)**	**91.37 ± 19.74**	**99.50 ± 25.30**	**0.009**	**0.347**
TG (mmol/L)	1.25(0.97,1.91)	1.37(0.99,2.04)	0.147	0.182
TC (mmol/L)	4.20(3.70,4.85)	4.41(3.62,5.14)	0.238	0.110
HDL-C (mmol/L)	1.22(1.08,1.52)	1.19(1.01,1.45)	0.360	0.112
LDL-C (mmol/L)	2.64 ± 0.91	2.56 ± 0.89	0.238	0.008
LDL-C/HDL-C	2.06 ± 0.80	2.24 ± 0.98	0.133	0.121
**lipoprotein(a)**	**177.00(96.50,323.00)**	**137.00(72.50,279.00)**	**0.048**	**0.225**
Fasting glucose (mmol/L)	5.38(4.73,6.35)	5.09(4.71,5.79)	0.075	0.155
Transferrin saturation, %	30.28 ± 12.35	34.74 ± 10.22	0.275	0.171
**Serum iron (µmol/L)**	**15.44 ± 6.80**	**18.08 ± 4.89**	**< 0.001**	**0.511**
**MoCA, score**	**23.00(21.00,24.50)**	**25.00(21.50,27.00)**	**< 0.001**	**0.811**
**AVLT, score**	**12.72 ± 5.32**	**16.60 ± 5.70**	**< 0.001**	**0.706**
**SDMT, score**	**23.17 ± 10.34**	**32.89 ± 12.75**	**< 0.001**	**0.825**
**SCWT, score**	**106.14 ± 38.58**	**85.0 ± 29.32**	**< 0.001**	**0.625**
**DST (inverted order)**	**4.00(3.50,7.00)**	**6.00(4.00,8.00)**	**< 0.001**	**0.581**

Abbreviations: WMH, White matter hyperintensities; BMI, body mass index; NLR, neutrophil-to-lymphocyte ratio; PLR platelet -to-lymphocyte ratio; WBC, white blood cells; MLR monocyte -to-lymphocyte ratio, SII systemic immune-inflammation index, (platelet count neutrophil count)/lymphocyte count; SIRI systemic inflammation response index, (neutrophil count ? monocyte count)/lymphocyte count; TG, triglyceride; TC, total cholesterol; HDL-C, high-density lipoprotein cholesterol; LDL-C, low-density lipoprotein cholesterol; MoCA, Montreal Cognitive Assessment; AVLT, Auditory Verbal Learning Test; SDMT, Symbol Digit Modalities Test; SCWT, Stroop Color-Word Test; DST, Digit Span Test

### Comparisons between the VCI and NCF groups

Compared to the NCF group, the VCI group exhibited a higher proportion of patients with sWMH (*P* < 0.001). Regarding serological markers, the VCI group demonstrated lower TG (*P* < 0.05, Cohen’s d = 0.215), LDL-C/HDL-C (*P* < 0.01, Cohen’s d = 0.511), LDL-C (*P* < 0.05, Cohen’s d = 0.297), and lymphocyte count (*P* < 0.01, Cohen’s d = 0.509). Conversely, PLR, MLR, SII, and lipoprotein(a) were higher in the VCI group compared to the NCF group (all *P* < 0.05). There were no significant differences observed in age, body mass index (BMI), sex, history of diabetes, alcohol consumption, smoking, hypertension, or other serological markers between the two groups (Table 2).

**Table 2:** Comparison of variables between the VCI and NCF groups

Characteristics	VCI (*n* = 132)	NCF (*n* = 125)	P value	Cohen’s d
Age (years)	64.67(61.00,70.00)	62.45(60.50.70.75)	0.115	0.325
BMI (kg/m^2^)	24.46 ± 3.46	24.78 ± 3.48	0.463	0.095
Gender, male, n (%)	77(58.3%)	88(70.4%)	0.106	
**WMH, Severe, n (%)**	**78(59.1%)**	**33(26.4%)**	**< 0.001**	
Hypertension, n (%)	93(70.5%)	86(68.9%)	0.604	
Diabetes, n (%)	39(29.5%)	43(34.4%)	0.878	
Smoking, n (%)	52(39.4%)	61(48.8%)	0.196	
Alcoholism, n (%)	41(31.1%)	40(32.0%)	0.964	
**Type of labor (Mental)**	**66(50.0%)**	**98(78.4%)**	**< 0.001**	
Lymphocyte (* 10 ^**9**^ **/L)**	**1.70 ± 0.52**	**1.99 ± 0.63**	**0.004**	**0.509**
Neutrophil (* 10^9^/L)	3.82(2.77,4.40)	4.03(2.83,4.64)	0.414	0.135
Platelet (* 10^9^/L)	211.20 ± 45.51	216.85 ± 58.47	0.393	0.108
NLR	2.17(1.59,2.86)	2.02(1.54,2.56)	0.104	0.262
**PLR**	**134.83 ± 53.33**	**114.68 ± 34.15**	**0.002**	**0.452**
WBC (* 10^9^/L)	6.12(5.01,7.19)	6.78(5.33,7.64)	0.098	0.212
**MLR**	**0.30(0.23,0.34)**	**0.28(0.21,0.32)**	**0.049**	**0.252**
**SII**	**528.38(315.56,564.65)**	**458.10(307.64,616.14)**	**0.046**	**0.258**
SIRI	1.18(0.71,1.45)	1.16(0.69,1.39)	0.819	0.059
**TG (mmol/L)**	**1.21(0.95,1.89)**	**1.36(1.04,2.08)**	**0.043**	**0.215**
TC (mmol/L)	5.17(4.87,3.61)	4.60(3.79,5.25)	0.157	0.128
HDL-C (mmol/L)	1.31(1.06,1.48)	1.57(1.04,1.44)	0.348	0.121
**LDL-C (mmol/L)**	**2.47 ± 0.86**	**2.73 ± 0.41**	**0.041**	**0.297**
**LDL-C/HDL-C**	**2.00 ± 0.79**	**2.43 ± 0.92**	**0.004**	**0.511**
Fasting glucose (mmol/L)	5.23(4.74,5.85)	5.31(4.82,5.95)	0.881	0.020
**lipoprotein(a)**	**232.75(196.50,319.00)**	**219.59(69.50,312.00)**	**0.032**	**0.262**

Abbreviations: WMH, White matter hyperintensities; BMI, body mass index; NLR, neutrophil-to-lymphocyte ratio; PLR platelet -to-lymphocyte ratio; WBC, white blood cells; MLR monocyte -to-lymphocyte ratio, SII systemic immune-inflammation index, (platelet count neutrophil count)/lymphocyte count; SIRI systemic inflammation response index, (neutrophil count ? monocyte count)/lymphocyte count; TG, triglyceride; TC, total cholesterol; HDL-C, high-density lipoprotein cholesterol; LDL-C, low-density lipoprotein cholesterol; MoCA, Montreal Cognitive Assessment; AVLT, Auditory Verbal Learning Test; SDMT, Symbol Digit Modalities Test; SCWT, Stroop Color-Word Test; DST, Digit Span Test

Logistic regression analysis adjusted for age and sex indicated that lymphocyte count (OR = 0.448, *P* < 0.001) and LDL-C/HDL-C (OR = 0.725, *P* = 0.028) were protective factors for cognitive function in patients with NICE. Meanwhile, WMHs(OR = 3.224, *P* < 0.001) and PLR(OR = 1.011, *P* = 0.002) were identified as a risk factor for cognitive decline in patients with NICE (Table 3).

**Table 3:** Variate of different cognitive groups with binary logistic regression

Variables	Adjusted* OR (95%CI)	P value
Lymphocyte (* 10 ^**9**^ **/L)**	**0.448(0.276,0.727)**	**< 0.001**
**PLR**	**1.011(1.004,1.018)**	**0.002**
MLR	1.751(0.065,46.833)	0.738
SII	1.001(0.998,1.003)	0.453
TG (mmol/L)	0.950(0.729,1.238)	0.513
**LDL-C/HDL-C**	**0.725(0.545,0.965)**	**0.028**
lipoprotein(a)	1.000(0.998,1.005)	0.864
**WMHs**	**3.224(1.841,5.667)**	**< 0.001**

**Abbreviations**: WMH, White matter hyperintensities; *: Adjusting for age and gender confounders; PLR platelet -to-lymphocyte ratio; MLR monocyte -to-lymphocyte ratio, SII systemic immune-inflammation index, (platelet count neutrophil count)/lymphocyte count; TG, triglyceride; HDL-C, high-density lipoprotein cholesterol; LDL-C, low-density lipoprotein cholesterol

### Diagnostic value of serological markers for VCI in different WMH groups

Among the 146 patients diagnosed with mild white matter hyperintensities (mWMH), 54 patients were classified under the VCI group. The VCI group exhibited significantly lower levels of LDL-C (*P* = 0.007), LDL-C/HDL-C (*P* = 0.009), lymphocyte count (*P* < 0.001), and neutrophil count (*P* = 0.025) compared to the NCF group. Conversely, the VCI group demonstrated higher levels of PLR (*P* = 0.035), MLR (*P* = 0.022), MLR (*P* = 0.022), and total cholesterol (*P* = 0.007) in comparison to the NCF group (Table 4). Logistic regression analysis of the aforementioned indicators, after adjusting for sex and age, revealed that lymphocyte count (OR = 0.415, *P* = 0.005) and LDL-C/HDL-C ratio (OR = 0.605, *P* = 0.015) were protective factors for cognitive function in patients with NICE (Table 5).

**Table 4:** Comparison of variables between the VCI and NCF groups in patients with mWMH

Characteristics	VCI (*n* = 54)	NCF (*n* = 92)	P value	Cohen’s d
Age (years)	61.26(57.00,66.00)	59.87(43.50,69.00)	0.413	0.143
BMI (kg/m^2^)	24.75 ± 3.44	24.78 ± 3.65	0.963	0.008
Gender, male, n (%)	23(42.6%)	62(67.4%)	0.301	
Hypertension, n (%)	37(68.5%)	59(64.11%)	0.719	
Diabetes, n (%)	14(25.9%)	28(30.4%)	0.695	
Smoking, n (%)	23(42.6%)	45(48.9%)	0.669	
Alcoholism, n (%)	19(35.1%)	29(31.5%)	0.614	
Lymphocyte (* 10 ^**9**^ **/L)**	**1.72 ± 0.56**	**2.09 ± 0.66**	**< 0.001**	**0.591**
Neutrophil (* 10 ^**9**^ **/L)**	**3.46(2.44,4.00)**	**4.00(2.80,4.84)**	**0.025**	**0.349**
Platelet (* 10^9^/L)	211.20 ± 45.51	216.85 ± 58.47	0.393	0.267
NLR	2.17(1.57,2.82)	2.02(1.51,2.59)	0.104	0.200
**PLR**	**131.67 ± 57.09**	**110.47 ± 34.6**	**0.035**	**0.467**
WBC (* 10^9^/L)	6.24(5.11,7.05)	6.68(5.23,7.58)	0.081	0.283
**MLR**	**0.30(0.23,0.34)**	**0.26(0.21,0.32)**	**0.022**	**0.366**
SII	482.81(267.15,565.65)	424.92(297.60,568.54)	0.331	0.117
SIRI	1.08(0.61,1.27)	1.07(0.64,1.35)	0.650	0.012
TG (mmol/L)	1.54(0.86,1.90)	1.86(1.06,2.13)	0.111	0.257
**TC (mmol/L)**	**6.22(3.56,4.81)**	**4.69(3.93,5.28)**	**0.007**	**0.178**
HDL-C (mmol/L)	1.33(1.02,2.03)	2.92(0.98,1.64)	0.696	0.125
**LDL-C (mmol/L)**	**2.35 ± 0.81**	**2.81 ± 0.91**	**0.007**	**0.514**
**LDL-C/HDL-C**	**1.94 ± 0.83**	**2.41 ± 1.02**	**0.009**	**0.507**
Fasting glucose (mmol/L)	5.04(4.64,5.75)	4.95(4.72,5.75)	0.403	0.062
lipoprotein(a)	212.63(96.50,328.00)	202.90 (66.50,251.00)	0.171	0.062

Abbreviations: WMH, White matter hyperintensities; BMI, body mass index; NLR, neutrophil-to-lymphocyte ratio; PLR platelet-to-lymphocyte ratio; WBC, white blood cells; MLR monocyte -to-lymphocyte ratio, SII systemic immune-inflammation index; SIRI systemic inflammation response index; TG, triglyceride; TC, total cholesterol; HDL-C, high-density lipoprotein cholesterol; LDL-C, low-density lipoprotein cholesterol; MoCA, Montreal Cognitive Assessment; AVLT, Auditory Verbal Learning Test; SDMT, Symbol Digit Modalities Test; SCWT, Stroop Color-Word Test; DST, Digit Span Test

**Table 5:** Variate of different cognitive mWMH subgroups with binary logistic regression

Variables	Adjusted* OR (95%CI)	P value
Lymphocyte (* 10 ^**9**^ **/L)**	**0.415(0.214,0.804)**	**0.005**
PLR	1.011(1.004,1.018)	0.432
MLR	1.751(0.065,46.833)	0.578
SII	1.001(0.998,1.003)	0.919
**LDL-C/HDL-C**	**0.605(0.403,0.908)**	**0.015**
TC (mmol/L)	0.950(0.729,1.238)	0.106

Abbreviations: WMH, White matter hyperintensities; *: Adjusting for age and gender confounders?PLR platelet -to-lymphocyte ratio; WBC, white blood cells; MLR monocyte -to-lymphocyte ratio, SII systemic immune-inflammation index; SIRI systemic inflammation response index; TG, triglyceride; TC, total cholesterol; HDL-C, high-density lipoprotein cholesterol; LDL-C, low-density lipoprotein cholesterol

**Fig. 1:**
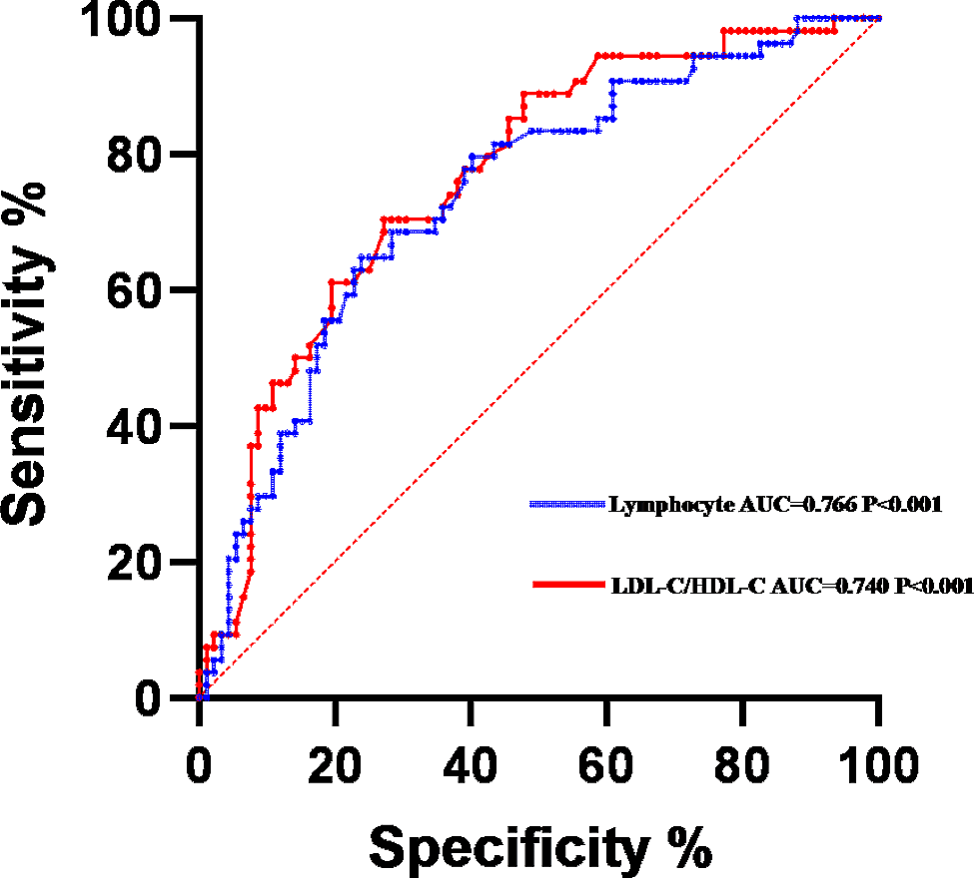
Diagnostic value of serological markers for vascular cognitive impairment in the mild white matter hyperintensity group

In addition, we conducted an analysis of receiver operating characteristic (ROC) curves to investigate the potential predictive and diagnostic significance of various indicators for VCI in patients with mWMH. Notably, lymphocytes exhibited the most substantial value. (AUC = 0.765, *P* < 0.001, 95% CI [0.687, 0.844], cutoff point: 1.83 × 10^9^/L, sensitivity: 70.37%, specificity: 72.83%). LDL-C/HDL-C also had acceptable value for the diagnosis of VCI (AUC = 0.740, *P* < 0.001, 95% CI [0.656, 0.822], cutoff point: 1.858mmol/L, sensitivity: 64.81%, specificity: 76.09%) (Fig. 1).

All of the gathered serologic indicators showed no significant difference between the VCI group and the NCF group among patients with sWMH (all *P* > 0.05).

## Discussion

The global prevalence of VCI has been steadily increasing in recent years [26]. VCI has been recognized to have a significant detrimental effect on the quality of life and prognostic outcomes of patients with CVD, underscoring the importance of identifying diagnostic indicators that can facilitate early clinical intervention [27]. In this research, we examined the relationships between serological markers, white matter hyperintensities, and VCI by analyzing the levels of serological markers in various WMH groups and their associations with different cognitive function levels in patients diagnosed with NICE.

We found that sWMH group scores on MOCA, AVLT, SDMT, SCWT, and DST (inverted order) are lower than those of mWMH group. This finding demonstrates a significant correlation between the severity of white matter injury in patients and the decline in executive function, memory, attention, and response inhibition, as supported by multiple studies [28, 29]. The precise mechanisms through which white matter hyperintensities contribute to VCI remain uncertain; however, abnormal lipid metabolism, neuroinflammation, endothelial dysfunction, and disruption of the blood-brain barrier are all potential influential factors [30, 31]. Previous research conducted by our team has shown that oligodendrocyte precursor cells, which play a crucial role in normal myelination, are susceptible to damage when exposed to mild ischemia and hypoxia. As a result of this damage, these cells release inflammatory factors that hinder their differentiation and maturation processes, ultimately leading to white matter injury and impacting cognitive function [32]. In our current study, we have observed that individuals with sWHM exhibit higher neutrophil count, NLR, MLR, PLR, SII and SIRI compared to those with mWMH. This finding suggests that certain inflammatory markers in peripheral blood could potentially serve as serological indicators of cerebral white matter injury in individuals with NICE patients.

Based on our investigation, patients diagnosed with VCI exhibited a diminished lymphocyte count, which emerged as an autonomous safeguarding element for cognitive functionality in patients with NICE. Previous research has substantiated the detrimental impact of peripheral inflammation on the integrity of the blood-brain barrier, leading to the infiltration of peripheral immune cells into the brain parenchyma and subsequent neuroinflammation [33]. Patients diagnosed with CVD who exhibit elevated inflammation-related markers in their peripheral blood are at a heightened risk of developing VCI. This increased susceptibility can be attributed to the continuous generation of proinflammatory agents subsequent to the activation of neuroinflammatory signaling pathways, which subsequently induce endothelial dysfunction, demyelination, glial stress, disruption of the blood-brain barrier, amyloidosis, and neuronal demise within the central nervous system. Consequently, these pathological processes culminate in cognitive dysfunction [34, 35]. Activation of the peripheral immune system leads to the adoption of a proinflammatory phenotype by immune cells, causing the release of proinflammatory factors into both the peripheral blood and brain. These factors have the potential to influence cognitive function [36, 37]. However, it is worth noting that there are also studies supporting the notion that lymphocytes play a protective role in cognitive function. For instance, Hou’s research suggests that microglia facilitate the recruitment of lymphocytes from the peripheral circulation to the central nervous system by releasing tumor necrosis factor-alpha in the event of blood-brain barrier disruption, thereby promoting the repairing of the blood-brain barrier [38]. In accordance with Shim’s study, it has been observed that the damage to myelin sheaths leads to the conversion of Th-1 lymphocytes, which promote inflammation, into the anti-inflammatory Th-2 type through apoptosis induced by growth factor-18 [39]. Additionally, Liesz’s research indicates that lymphocytes can hinder the spread of neuroinflammation by releasing interleukin-10, a cytokine known for its neuroprotective properties [40]. Consequently, this facilitates the restoration of cognitive function by enabling the repair of damaged cerebral white matter [41]. Based on the findings of the study, lymphocyte-mediated inflammatory responses can predict and potentially stop cognitive decline in NICE patients.

Our findings indicated that patients with VCI had lower LDL-C/HDL-C, and LDL-C/HDL-C was an independent protective factor for cognitive function in patients with NICE. Previous research has shown that therapeutic interventions targeting the reduction of LDL-C levels have been associated with cognitive decline in patients, potentially due to the inhibition of myelin synthesis and restoration within the central nervous system [42, 43]. The study by Zhu proposes that LDL-C forms complexes with enzymes such as lipoprotein-associated phospholipase A2 and superoxide dismutase, thereby exerting antioxidative and anti-inflammatory effects to enhance cognitive function [44]. In addition, a population-based study conducted in Shanghai, China, revealed a negative correlation between elevated LDL levels and cognitive dysfunction in individuals with fewer vascular risk factors [45]. This suggests that cholesterol plays a crucial role in neuronal components, and low cholesterol levels may impede dendritic growth, synapse formation, contribute to neurodegeneration, and impair cognitive function [46]. However, some researchers have proposed contrasting conclusions regarding the relationship between LDL-C and cognition. These studies suggest that elevated cholesterol levels increase the risk of cognitive impairment, potentially due to the development of atherosclerosis and chronic cerebral hypoperfusion, both of which indirectly contribute to cognitive dysfunction [42, 47, 48]. A longitudinal study conducted on a healthy population by Hua found that low LDL-C levels were associated with a slower rate of cognitive decline [49]. This association may be explained by the fact that, under normal circumstances, differences in plasma cholesterol concentrations do not readily penetrate the blood-brain barrier, and cholesterol synthesized by astrocytes and oligodendrocytes fulfills the neurological activity requirements [50]. Additionally, research has shown that plasma cholesterol can influence lipid metabolism within the central nervous system when the integrity of the blood-brain barrier is compromised during a cerebrovascular event, potentially explaining the significant controversies surrounding the relationship between lipids and cognition [51, 52]. The current results indicate that peripheral plasma LDL-C / HDL-C as well as potential lipid metabolic pathways are strongly associated with vascular cognitive impairment in patients with NICE.

The results of this study indicate that lymphocytes and LDL-C/HDL-C are protective factors for cognitive function in patients with NICE who have mWMH. At the same time, ROC curves based on the presence or absence of VCI indicated that lymphocyte and LDL-C/HDL-C had good value in the diagnosis of VCI in patients with mWMH, with lymphocyte count representing the most diagnostic factor. However, no serological markers exhibited significant role in cognitive function in patients with sWMH. The research of Sliz and Zhang posits that in the initial stages of white matter damage, myelin undergoes self-repair, over 70% of myelin’s composition is lipid, with cholesterol maintaining the stability of lipid transport proteins and myelin structural proteins, this repair activity significantly declines in the later stages of white matter damage [53, 54]. Concurrently, other studies suggest that compared to severe white matter damage, individuals with mild white matter damage do not entirely lose central nervous system signal transmission and metabolic functions at all levels [28, 55]. Nevertheless, the findings of Zeng contradict ours, indicating that peripheral blood biological markers are more valuable in severe white matter damage patients [56]. This may be due to the near-complete loss of myelin protective effects and severe impairment of endothelial cell function in the later stages of white matter damage, leading to a more thorough breakdown of the blood-brain barrier. the changes in peripheral blood biological markers better align with those in the central nervous system [29, 57]. Our study suggests that lymphocyte count and LDL-C/HDL-C have significant predictive and evaluative value for assessing the severity of VCI in patients with mild white matter damage, with lymphocyte count being the most valuable.

The present investigation possesses specific constraints that necessitate acknowledgment. Firstly, the cross-sectional design employed in the study prohibits any establishment of causality. Consequently, longitudinal studies are imperative to authenticate our findings. Secondly, the evaluation of all serological markers occurred solely on a single occasion, potentially introducing inaccuracies in laboratory measurements and consequently impacting the accuracy of the data. Lastly, the sample size was relatively modest, potentially introducing bias into the outcomes.

In conclusion, our research findings suggest that lymphocyte count and the LDL-C/HDL-C are significant independent factors that protect cognitive function in patients with NICE. Additionally, our analysis highlights the potential of neutrophil count, NLR, MLR, PLR, SII and SIRI as a serological marker for white matter injury. Specifically, lymphocytes and the LDL-C/HDL-C ratio were observed to be protective factors for cognitive function in patients with NICE who exhibited white matter hyperintensities (mWMH). Furthermore, lymphocyte count and the LDL-C/HDL-C ratio demonstrated promising diagnostic values for vascular cognitive impairment (VCI) in patients with mWMH, with lymphocyte count showing the highest diagnostic value. However, no valuable serological markers were identified in the sWMH group. In the future, prospective studies with larger sample sizes are needed to further explore the associations among VCI, serologic inflammatory markers, and WMH.

## Data Availability

The data presented in this study are available on request from the corresponding author.
